# The effect of estrogen signaling modulation on female‐specific microglial heterogeneity in the mouse hippocampus

**DOI:** 10.1002/alz.090388

**Published:** 2025-01-03

**Authors:** Sunghwan Ko

**Affiliations:** ^1^ University of Oklahoma Health Sciences Center, Oklahoma City, OK USA

## Abstract

**Background:**

Estrogens, such as 17β‐estradiol, are the primary female sex hormones predominantly synthesized by mature ovarian follicular cells. The natural exhaustion of ovarian follicular cells during menopause causes a rapid decline in endogenous estrogen levels. This decline in estrogen levels is associated with an increase in chronic, age‐related pathologies, including inflammation in the brain. Studies suggest that estrogens can temper inflammatory processes in the brain by activating estrogen receptor α (ERα), a nuclear transcription factor expressed by microglia, the brain’s primary resident macrophage. However, the pathways responsible for estrogens’ anti‐inflammatory effects in microglia remain unresolved. Here, we hypothesize that estrogen signaling through microglial ERα suppresses pro‐inflammatory signaling pathways. We believe that ablation of endogenous estrogens during menopause induces neuroinflammation and promotes the transition of microglia to activated phenotypes, contributing to various brain diseases, including Alzheimer’s disease (AD).

**Method:**

To test the effect of estrogenic depletion on microglial heterogeneity in mice, we: 1) knocked out ERα, 2) depleted endogenous estrogens with 4‐vinylcyclohexene diepoxide (VCD) treatment, and 3) compared to untreated female C57BL/6 controls. We then examined the changes in hippocampal microglial phenotypic states using 10X single‐cell transcriptomic sequencing (scRNA‐seq).

**Result:**

scRNA‐seq data were quality filtered, and unbiased clustering analyses (i.e., UMAP) identified five hippocampal microglial subtypes. Interestingly, the expression of specific microglial phenotypic state (stage 1 disease‐associated microglia) was increased upon ablation of endogenous estrogen production by VCD or knockout of ERα.

**Conclusion:**

Such skewing of microglia towards reactive phenotype suggests that loss of estrogen‐ ERα signaling may encourage “priming” of microglia to transition to the stage 1 DAM phenotype, which is then more susceptible to becoming “reactive” DAMs (stage 2 DAMs).

